# Genomic Distribution and Divergence of Levansucrase-Coding Genes in *Pseudomonas syringae*

**DOI:** 10.3390/genes3010115

**Published:** 2012-02-10

**Authors:** Abhishek Srivastava, Nehaya Al-Karablieh, Shaunak Khandekar, Arifa Sharmin, Helge Weingart, Matthias S. Ullrich

**Affiliations:** School of Engineering and Science, Jacobs University Bremen, Bremen 28759, Germany; E-Mails: a.srivastava@jacobs-university.de (A.S.); n.alkarablieh@jacobs-university.de (N.A.-K), s.khandekar@jacobs-university.de (S.K.); a.sharmin@jacobs-university.de (A.S.); h.weingart@jacobs-university.de (H.W.)

**Keywords:** levansucrase, phage-associated promoter element, *Pseudomonas syringae*, *Erwinia amylovora*, pro-phage

## Abstract

In the plant pathogenic bacterium, *Pseudomonas syringae*, the exopolysaccharide levan is synthesized by extracellular levansucrase (Lsc), which is encoded by two conserved 1,296-bp genes termed *lscB* and *lscC* in *P. syringae* strain PG4180. A third gene, *lscA*, is homologous to the 1,248-bp *lsc* gene of the bacterium *Erwinia amylovora*, causing fire blight. However, *lscA* is not expressed in *P. syringae* strain PG4180. Herein, PG4180 *lscA* was shown to be expressed from its native promoter in the Lsc-deficient *E. amylovora* mutant, Ea7/74-LS6, suggesting that *lscA* might be closely related to the *E. amylovora lsc* gene. Nucleotide sequence analysis revealed that *lscB and lscC* homologs in several *P. syringae* strains are part of a highly conserved 1.8-kb region containing the ORF, flanked by 450-452-bp and 49-51-bp up- and downstream sequences, respectively. Interestingly, the 450-452-bp upstream sequence, along with the initial 48-bp ORF sequence encoding for the N-terminal 16 amino acid residues of Lsc, were found to be highly similar to the respective sequence of a putatively prophage-borne glycosyl hydrolase-encoding gene in several *P. syringae* genomes. Minimal promoter regions of *lscB* and *lscC* were mapped in PG4180 by deletion analysis and were found to be located in similar positions upstream of *lsc* genes in three *P. syringae* genomes. Thus, a putative 498-500-bp promoter element was identified, which possesses the prophage-associated *com* gene and DNA encoding common N-terminal sequences of all 1,296-bp Lsc and two glycosyl hydrolases. Since the gene product of the non-expressed 1,248-bp *lscA* is lacking this conserved N-terminal region but is otherwise highly homologous to those of *lscB* and *lscC*, it was concluded that *lscA* might have been the ancestral *lsc* gene in *E. amylovora* and *P. syringae*. Our data indicated that its highly expressed paralogs in *P. syringae* are probably derived from subsequent recombination events initiated by insertion of the 498-500-bp promoter element, described herein, containing a translational start site.

## 1. Introduction

The gram-negative phytopathogenic bacterium, *Pseudomonas syringae*, is classified into 51 pathovars based on distinct host specificities [1]. These bacteria produce various extracellular polysaccharides (EPSs). In *P. syringae*, *Erwinia amylovora*, and several other bacterial species, the EPS levan is synthesized from sucrose by the extracellular enzyme levansucrase (Lsc; EC 2.4.1.10), which is a member of the glycosyl hydrolase 68 family. Levan is a high-molecular β-(2,6)-polyfructan with extensivebranching through β-(2,1)-linkages [2]. 

*P. syringae* pv. glycinea PG4180 causes bacterial blight on soybean plants and has been used as a model strain for levan formation [2,3,4]. Full genome sequences of three other *P. syringae* strains are available: pv. phaseolicola 1448A, pv. syringae B728a, and pv. tomato DC3000. Partial shot-gun genome sequences of several other *P. syringae* strains are available on the NCBI’s Genbank website. Comparison of genome sequences in *P. syringae* strains revealed the presence of 2–3 copies of the *lsc* gene in all of the analyzed strains [3,4,5,6,7,8,9]. In previous years extensive progress has been made in terms of the heterologous expression and the protein and polymer characterization of *lsc* genes from *P. syringae* [10,11,12].

Bacterial genomes are comprised of core and flexible components. Core genomes include genes essential for the survival of the organism, such as e.g., 365 housekeeping genes in *P. syringae* [6]. Phylogenetic comparison of several housekeeping genes allowed classification of *P. syringae* strains into four monophyletic groups, where pv. tomato DC3000 and T1 belonged to group 1, pv. syringae B728a to group 2, and pv. phaseolicola 1448A as well as pv. glycinea PG4180 to group 3 [13]. Flexible genomic components comprise genes important for adaptation to specific ecological niches or specific growth conditions such as e.g., virulence-associated genes, resistance genes, or mobile genetic elements like phage-borne genes, plasmids, conjugative transposons, or insertion sequence (IS) elements [13]. 

In contrast to any other investigated levan-forming bacteria, multiple copies of *lsc* have been reported for *P. syringae* strains. However, a reasonable explanation for the occurrence of multiple *lsc* copies in *P. syringae* genomes is missing. Recently, genomic data of plant-pathogenic and plant-associated bacteria has emerged rapidly and a close observation indicates the presence of more than one copy of *lsc* genes present in *P. syringae* strains [3,4,5,6,7,8,9]. The *lsc* alleles in PG4180 were termed *lscA*, *lscB*, and *lscC* [3] while the corresponding genes in DC3000 and T1 were termed *lsc2*, *lsc3*, and *lsc1* [5,8]. Two distinguishable variants of *lsc* were observed: *lscA* and *lsc2* are 1,248 bp in length while *lscB, lsc3*, *lscC*, and lsc*1* comprise 1,296 bp. Strain B728a possesses only one 1,296-bp and one 1,248-bp *lsc* allele [7]. For easier understanding, herein the 1,248-bp *lsc* gene variants were designated as ‘variant A’ while the 1,296-bp *lsc* alleles were termed ‘variant BC’. 

*E. amylovora* Ea7/74, which causes fire blight on rosaceous plants, possesses a single variant A homolog [2,14]. Mutation of *lsc* in Ea7/74 led to a levan-negative phenotype [15]. Previously, variant A from PG4180 was shown to be not expressed under various conditions tested in its native host. Mutation of variant A in PG4180 still rendered the mutant levan-positive [3]. Only simultaneous mutation of *lscB* and *lscC* yielded the levan-negative mutant, PG4180.M6 [3]. Furthermore, *lscA* of PG4180 is not expressed from its native promoter in *Escherichia coli*, a close enterobacterial relative of *E. amylovora* [2,3].

In the current study, the cryptic PG4180 variant A *lsc* gene was expressed from its native promoter in the *E. amylovora lsc*-negative mutant, Ea7/74-LS6, demonstrating that the *E. amylovora* genetic background was sufficient for its expression. A comparative bioinformatics approach was used to analyze the upstream and downstream sequences of variant BC alleles in five *P. syringae* strains, giving rise to an interesting model on how *lsc* genes might have evolved and been distributed among *P. syringae* pathovars. Furthermore, it was determined that variant BC alleles might be expressed from a newly defined phage-associated promoter element (PAPE).

## 2. Results and Discussion

### 2.1. Heterologous Expression of lscA in *E. amylovora*

**Figure 1:**
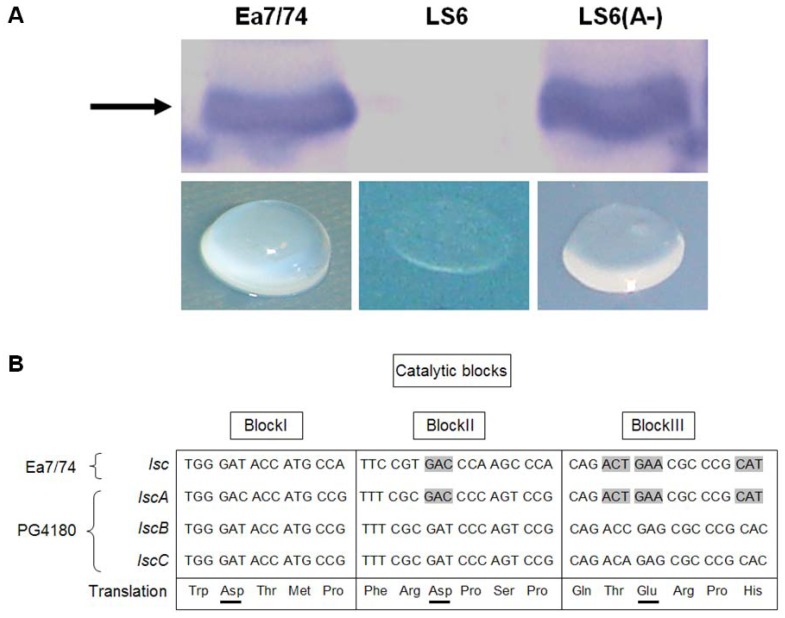
(**A**) Western blot detection of extra-cellular levansucrase (Lsc) of *E. amylovora* Ea7/74 and LscA of *P. syringae* pv. glycinea PG4180 expressed in Ea7/74-LS6 (*lsc* deficient mutant). LS6 (*lscA*) harbored PG4180 *lscA* in opposite orientation to the vector-borne P*_lac_* promoter. The arrow represents the signal for the 55-kDa Lsc proteins. The lower panel shows the levan formation assay for cell-free concentrated culture supernatants on water agar plates supplemented with 5% sucrose. The sample was incubated at 18 °C for one week on a water agar plate with 1.5% agar content (**B**) Comparison of the predicted catalytic blocks present in the active sites of PG4180 variant A and BC with that of *E. amylovora* Ea7/74 Lsc. Residues Asp, Asp, Glu in blocks I, II, and III are the predicted key residues involved in activity of the enzyme. Lsc of Ea7/74 and PG4180 variant A share the same codon usage for Block II Asp and Block III Glu.

Previously, it had been shown that *lscA* was not expressed in PG4180 while variant BC alleles were functional [3]. When *lscA* of PG4180 along with its 940-bps upstream sequence was introduced to the *lsc*-negative *E. amylovora* mutant Ea7/74-LS6 [15], the resulting transconjugant showed levan formation on agar plates supplemented with 5% sucrose, in contrast to mutant Ea7/74-LS6 (Data not shown). Since *lscA* was placed in the opposite direction to vector-borne promoters of the plasmid, this result indicated *lscA* expression from its native promoter. To substantiate this, Western blot analysis with Lsc-specific antiserum and protein extracts of Ea7/74 wild type, its *lsc*-deficient mutant, and the Ea7/74-LS6 transconjugant carrying *lscA* was conducted, revealing a clear signal for Lsc in the transconjugant (Figure 1A). Concentrated cell-free supernatants of the Ea7/74 derivatives were spotted on water agar containing 5% sucrose revealing levan formation in the transconjugant thus confirming heterologous *lscA* expression (Figure 1A).

### 2.2. Nucleotide Sequence Comparison of Variant A *lsc* Genes

**Figure A1:**
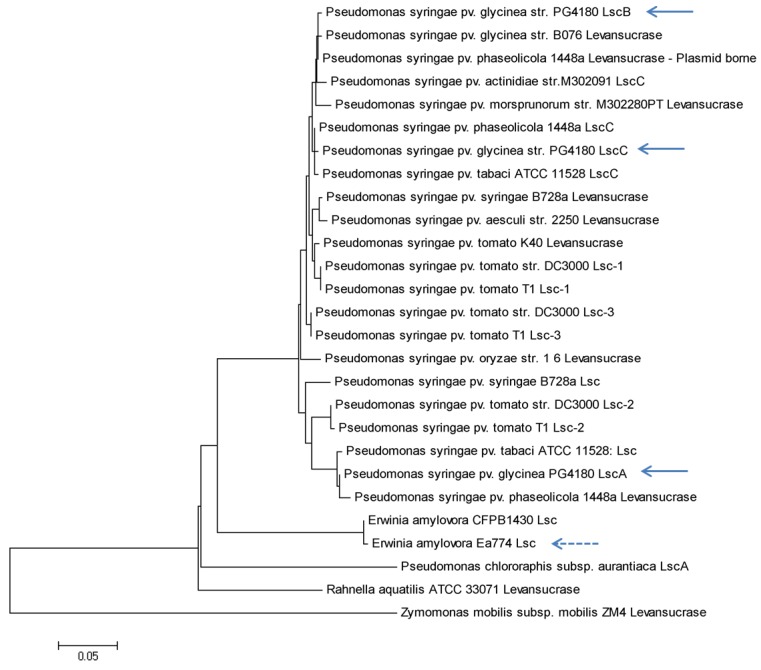
Neighbor-Joining phylogenetic tree generated by Mega 5.05 for known Lsc amino acid sequences of *P. syringae* strains, *Erwinia amylovora*, *Rahnella aquatilis* and *Zymomonas mobilis*. The blue arrows represent Lsc in PG4180 while the dashed arrow represents Lsc of Ea7/74.

PG4180 LscA shows 87.5% identity at the amino acid level with variant BC enzymes and shares 75.9% identity to Lsc of Ea7/74. It was speculated that the common ancestor of PG4180 genes *lscA*, *lscB*, and *lscC* might be related to the *lsc* gene of *E. amylovora*, and that all three genes present in *P. syringae* might have diverged from this common ancestor. This hypothesis was supported by a phylogenetic analysis of all available protein sequences of Lsc’s built by the Neighbor-Joining method (Appendix Figure A1). Variant A alleles of all *P. syringae* strains were found to be clustered closer to the Lsc’s from *Enterobacteriaceae* including *E. amylovora* Ea7/74 as opposed to variant BC alleles. Aside of variant A alleles being present in single copy in several *P. syringae* strains and in *E. amylovora,* this gene variant was also found in single copy in other enterobacterial species such as *Erwinia tasmaniensis* and *Rahnella aquatilis* ATCC33071 (Table 1). Since these bacterial species are commonly found to be associated with host plant species such as soybeans [16,17], *P. syringae*, *E. amylovora*, and *R. aquatilis* might have exchanged genetic information during cohabitation and *lscA* can be considered as an example of such horizontal gene transfer.

**Table 1:** Distribution of variant A and variant BC alleles in *Pseudomonadaceae* and *Enterobacteriaceae*.

		variant A (1,248 bp)			variant BC (1,296 bp)			
	Pathovar, strain	Gene name	Locus tag	Genomic location	Gene name	Locus tag	Genomic location	References
*Pseudomonadaceae*								
*Pseudomonas syringae*	glycinea PG4180	*lscA*	**-**	Chr.	*lscB*	**-**	Plasmid	[3]
					*lscC*	**-**	Chr.	
	glycinea B076	*levansucrase*	PsgB076_10300	?	*lscC*	PsgB076_00457	?	[9]
	glycinea race 4	*levansucrase*	PsgRace4_15609	?	*lscC*	PsgRace4_03819	?	[9]
	phaseolicola 1448A	*levansucrase*	PSPPH_2074	Chr.	*levansucrase*	PSPPH_A0027	Plasmid	[6]
					*lscC*	PSPPH_4994	Chr.	
	syringae B728a	*levansucrase*	Psyr_2103	Chr.	*levansucrase*	Psyr_0754	Chr.	[7]
	actinidiae M302091	**-**			*lscC*	PSYAC_19498	?	[42]
	aesculi NCPPB3681	*levansucrase*	PsyrpaN_010100019209	?	**-**			[43]
	lachrymans M302278PT	*levansucrase*	PLA107_25445	?	**-**			[43]
	morsprunorum M302280PT	**-**			*levansucrase*	PSYMP_24576	?	[43]
	tabaci ATCC 11528	*levansucrase*	PSYTB_12850	?	*lscC*	PsyrptA_020100005135	?	[42,43]
	tomato DC3000	*lsc-2*	PSPTO_2305	Chr.	*lsc-3*	PSPTO_A0032	Plasmid	[5]
					*lsc-1*	PSPTO_1453	Chr.	
	tomato T1	*lsc-2*	PSPTOT1_4965	Chr.	*lsc-3*	PSPTOT1_4913	?	[8]
					*lsc-1*	PSPTOT1_1070	Chr.	
	tomato K40	**-**			*levansucrase*	PsyrptK_010100027584	?	Vinatzer *et al.* (unpublished) Genbank
	tomato NCPPB 1108	*levansucrase*	PsyrptN_010100027628	?	**-**			Vinatzer *et al.* (ubpublished) Genbank
*Enterobacteriaceae*								
*Erwinia amylovora*	Ea7/74	*Lsc*	**-**	Chr.	**-**			[15]
	CFPB1430	*Lsc*	**-**	Chr.	**-**			[44]
*Erwinia tasmaniensis*	Et1/99	*Lsc*	ETA_34670	Chr.	**-**			[45]
*Rahnella aquatilis*	ATCC33071	*lsrA*	**-**	Chr.	**-**			[46]

*lsc*: levansucrase, Chr.: Chromosomal location.

It had been reported that three acidic residues are highly conserved among members of the Glycosyl hydrolase families 32, 43, 62, and 68 at the catalytic active sites termed as block I (Asp/Glu), block II (Asp) and block III (Glu) [12,18,19]. Therefore, conserved regions within the catalytic blocks of variant A and variant BC were compared for Ea7/74 and PG4180 (Figure 1B). The catalytic centers Asp (block II) and Glu (block III) shared a common codon usage for these nucleotide sequences with both organisms (www.kazusa.or.jp/codon/). This further suggested that *lscA* in PG4180 and *lsc* of Ea7/74 might share similar regulatory features particularly present in *E. amylovora*. 

### 2.3. Nucleotide Sequence Comparison of Variant BC *lsc* Genes

In contrast to variant A genes, variant BC *lsc* alleles are found only in *P. syringae* pathovars (Table 1, Appendix Figure A1). The coding sequences of all variant BC genes exhibited 94.0% identity at the nucleotide sequence level and 98.1 to 99.8% similarity at the protein sequence level, demonstrating a high degree of conservation among otherwise variable *P. syringae* pathovars [13,20]. 

**Figure 2:**
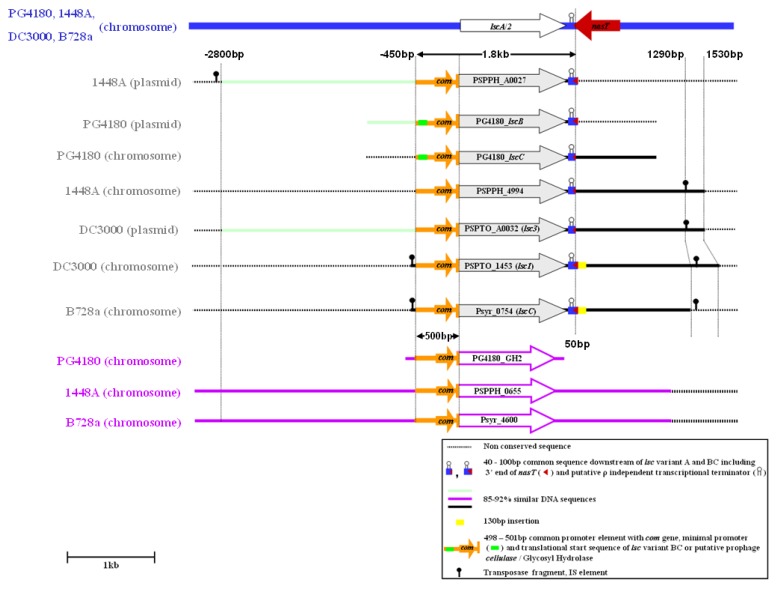
Genetic map showing variant BC alleles and their surrounding sequences in *P. syringae* pathovars represented by PG4180, 1448A, DC3000 and B728a. The 1.8-kb conserved nucleotide sequence contains 1.296-bp *lsc* coding sequence, 450 bp up-, and 49–51 bp downstream sequences of variant BC. The 500-bp conserved sequence represents the phage-associated promoter element (PAPE) linked with variant BC and the putative pro-phage-borne glycosyl hydrolase genes with 48 bp conserved N-termini of the coding sequences. PSPPH_0655 and Psyr_4600 are putative glycosyl hydrolase genes in 1448A and B728a, respectively. The PAPE contains the promoter of *lsc* and *com* genes. Variant BC comparison in their downstream sequences showed a different length of nucleotide conservation. The 25–100 bp sequences downstream of *lscB/C* stop codon show conservation with a 75-bp *lscA* downstream sequence and the 3’ end of the *nasT* gene. Variant A is depicted devoid of the 48-bp conserved sequence which is always associated with variant BC. Minus (‘−’) values depict upstream sequences to the translational start codon of *lsc* and positive values represent the nucleotide sequences downstream of *lsc* translational stop codon.

**Figure 3:**
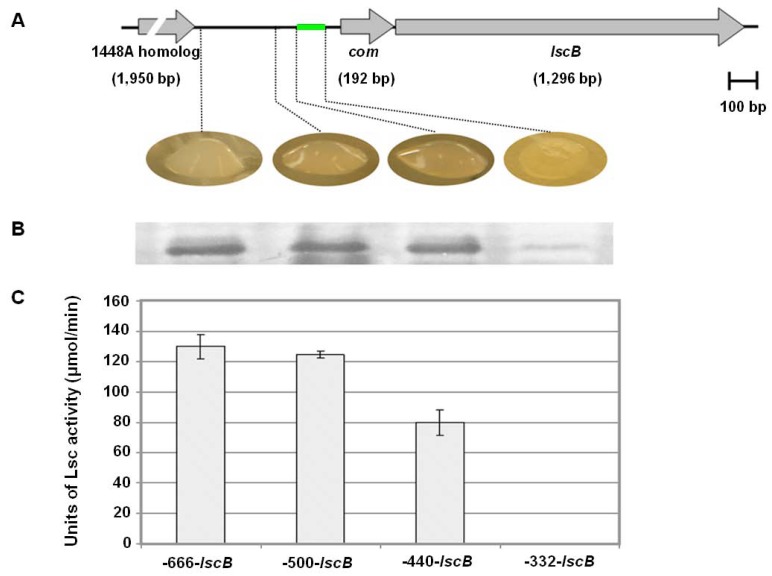
Nested deletion analysis of the *lscB* upstream sequence. (**A**) Schematic presentation of *lscB* and its upstream sequence. (**■**) represents the promoter region of *lscB*. Levan phenotypes after complementation of mutant PG4180.M6 with different deletion constructs are provided below. (**B**) Western blot analysis of 30-fold concentrated cell-free supernatants of PG4180.M6 complemented with deletion constructs using Lsc-specific antiserum. (**C**) Lsc activities in 1 mL of cell-free supernatants. Cells were grown at 18 °C and harvested at OD_600_ of 1.5 to 2.0. Data represent average values with standard deviation from three independent experiments each with three replicates.

**Figure A2:**
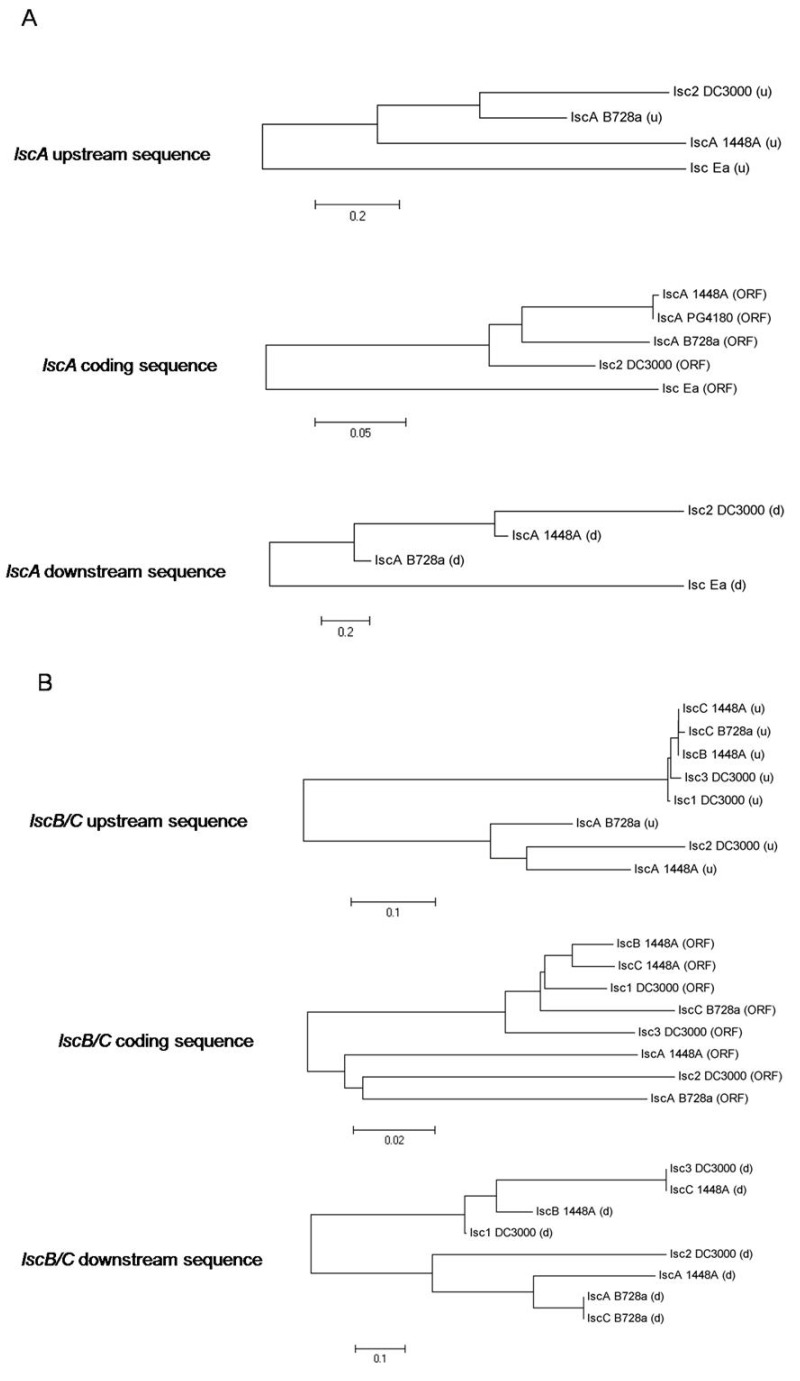
Phylogenetic dendrograms of variant BC *lsc* genes and their up- and downstream sequences.

**Figure A3:**
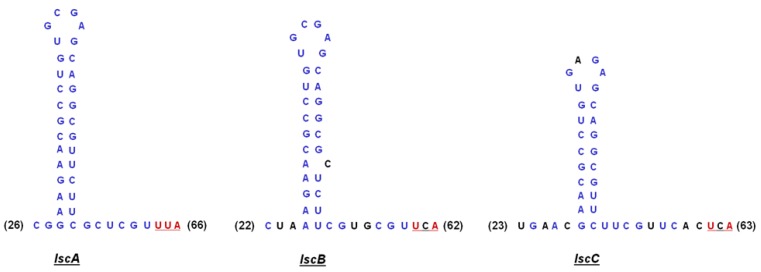
Schematic depiction of predicted stem loop structures at the mRNA level for *lscA, lscB*, and *lscC* transcripts. The poly U runs suggest ρ-independent transcriptional termination. Blue nucleotide letters were found identical to those of the *lscA* downstream sequence. Red letters symbolize the position of the stop codon of *nasT* gene in case of *lscA*.

**Figure A4:**
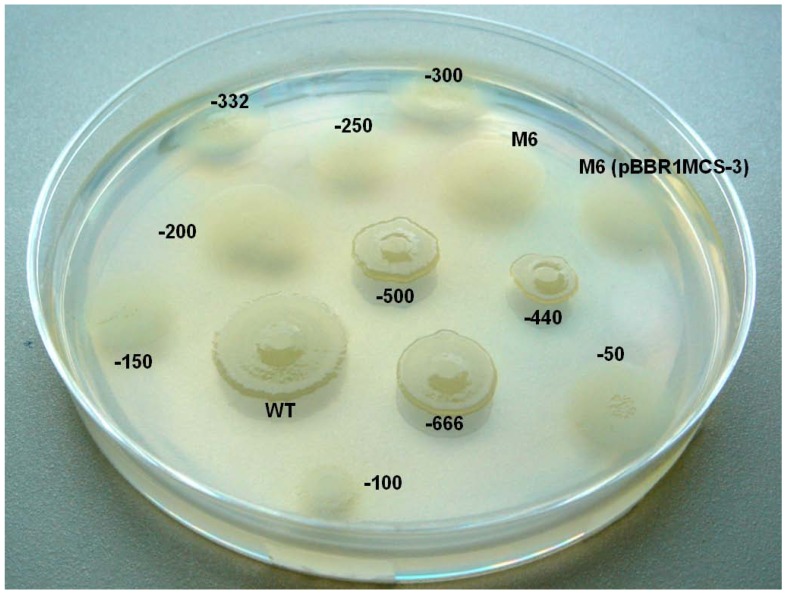
Phenotype of the *lscBC*-deficient mutant PG4180.M6 complemented with various *lscB* deletion constructs (ranging from -50 to -666-bp upstream of the translational start of *lscB* gene) and grown on MG agar supplemented with 5% sucrose. PG4180.M6 (pBBR1MCS) and PG4180.M6 served as negative controls. WT represents PG4180.WT serving as the positive control.

Comparison of the up- and downstream sequences of known variant BC alleles interestingly revealed a common ~1.8-kb highly conserved nucleotide sequence with an average of 87.3% identity (Figure 2, Appendix Figure A2). This conserved region comprises 450 to 452 bp of upstream sequence, 1,296 bp of the *lsc* ORF and 49-51 bp of downstream sequence, possibly involved in the formation of stem-loop structure for ρ-independent transcriptional termination (Appendix Figure A3). To map the promoter element of variant BC genes, a nested deletion analysis of the *lscB* upstream sequences ranging from -666 to -50 bp of the respective *lsc* translational start (TS) was conducted (Figure 3). For this, the *lscB*- and *lscC*-deficient mutant PG4180.M6 was complemented with various plasmid-borne deletion constructs and the phenotypes of the transconjugants were analyzed with respect to levan production (Appendix Figure A4). Deletion constructs ending 5’ at position −440-bp upstream of the TS and any larger upstream sequence fully complemented the mutant PG4180.M6 with respect to levan formation. In contrast, the deletion construct ending at position −332-bp did not complement this mutant, thereby identifying the minimal promoter required for *lscB* expression (Figure 3). The experiment was repeated with respective deletion constructs for *lscC* giving identical results (Data not shown). Since nucleotides 450–452 bp upstream of variant BC ORFs are highly conserved in all four *P. syringae* strains analyzed (Figure 2, Appendix Figure A2), it may be speculated that the minimal promoter sequences of the variant BC alleles of the other *P. syringae* pathovars not experimentally studied are similar to those of strain PG4180. Nucleotide sequences flanking the conserved ~1.8 kb region varied considerably among the strains investigated, with the exception of two plasmid-borne variant BC alleles in strains DC3000 and 1448A, for which 93% of the nucleotides up to −2,800 bp with respect to the translational start sites were identical (Figure 2). This 2,800-bp upstream sequence of plasmid-borne variant BC genes is bordered by a truncated transposase gene resembling that of transposon ISPsy16 [6] (Data not shown).

### 2.4. Downstream Sequence Comparison of Variant BC *lsc* Alleles

Aside from the 49–51-bp highly conserved DNA sequences, downstream of the translational stop of variant BC genes, the nucleotide sequences of those alleles further downstream exhibited ~87% identity until +1,530 bp with respect to *lsc* translational stop codon, except for *lscC* of B728a and *lsc-1* of DC3000, which both had an additional 130-bp 85% similar downstream sequence (Figure 2, Appendix Figure A2). Since strain B728a lacks any native plasmid [7] but possesses conserved sequences surrounding *lscC* (−478 bp with respect to translational start and +1,420 bp with respect to the translational stop of the *lsc* gene) to that of DC3000 *lsc-1* (Figure 2), it was speculated that *lsc-1* of the pv. tomato strain might have been associated with the phylogenetic source of *lscC* in B728a. 

**Figure 4:**
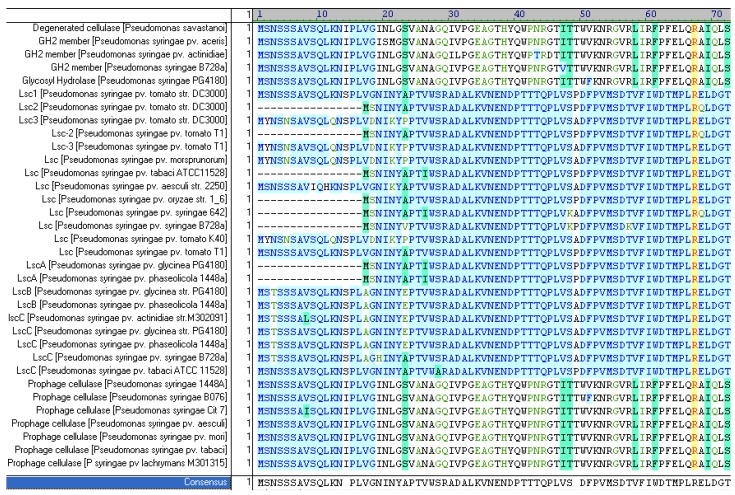
Amino acid sequence alignment of N-termini of predicted gene products (variant A, variant BC, putative pro-phage-borne glycosyl hydrolase). A 16 residue-spanning conserved sequence in variant BC and the glycosyl hydrolase is missing in variant A. Color coding: blue—conservative residue, green—block of similar residues, yellow/orange—identical residues, white/green—weakly similar residues, black—non-similar residues.

Nucleotide sequence alignments revealed sequences downstream of variant BC alleles with significant similarities to sequences of transposases (www.pseudomonas.com). These could be considered as presumable evolutionary scars of transposable elements involved in the course of variant BC allele distribution in *P. syringae* (Figure 2). In the genome of strain B728a a sequence showing 92% identity with transposase orfA of ISPsy24 [6] was located 1,450–1,488 bp downstream of the *lscC* stop codon. Interestingly, the sequence of 1,420 bp downstream of B728a *lscC* coincided with the 1,290-bp downstream sequences of *lscC* in strains 1448A and PG4180 as well as of *lsc3* in strains DC3000 and T1 (Appendix Figure A2). This finding further supported the hypothesis that *lscC* of strain B728a might be derived from a similar source to that of *lsc-1* from strain DC3000. Previously, studies on *E. coli* transposon IS911 showed that production of OrfA either *in cis* or *in trans* stimulated production of excised circular transposon copies, suitable for intermolecular transposition into a plasmid target [21]. Previously, IS elements had been predicted to be involved in the horizontal transmission of avirulence genes and the coronatine gene cluster in *P. syringae* [22,23]. In a comparative study, *lsc-1* (chromosomal) was located on the variable region (VR) 38 and supposed to be in a hotspot zone [24]. It has been reported that the same location is functionally classified as a lineage-specific region (LSR) *i.e.*, virulence type LSR no. 14, in the DC3000 chromosome. The majority of DC3000-specific genes were suggested to be linked with lateral gene transfer events and responsible for the fitness as well as adaptation to the environment. Such LSRs were also predicted to help in utilizing plant-derived energy sources, e.g., sucrose [6].

Interestingly, the 25-100-bp downstream sequences of *lscC* in strain B728a, of *lscB* in strains 1448A and PG4180, and of *lsc-1* in strain DC3000, showed an overall 72% sequence identity with the 75-bp downstream sequences of variant A *lsc* alleles. Forty nucleotides of this 75-bp sequence were found to be conserved in the 3’-end of the *nasT* gene coding for a response regulator protein, required for expression of nitrite-nitrate reductase genes (Figure 2) suggesting but not proving a phylogenetic link between a putative initial insertion of an ancestral variant A allele to *P. syringae*, which later diverted into several chromosomal or plasmid-borne variant BC alleles. 

### 2.5. Upstream Sequence Comparison of Variant BC *lsc* Alleles and a Putative Prophage-Borne Glycosyl Hydrolase Gene

A genome-wide comparison of the 450–452-bp upstream sequences of variant BC *lsc* alleles with the genomic sequences of strains 1448A, DC3000, and B728a, respectively, revealed 452-bp and 70% conserved sequences upstream of two putative family 2 glycosyl hydrolase genes located within the sequences of pro-phage PSPPH01 of strain 1448A and pro-phage GH5 of strain B728A (Table 2). The sequence focused-on herein was also found to be associated with a putative bacteriocin gene in DC3000 although to a lesser extent (Table 2). In *Klebsiella* sp., a gene cluster, required for production of the bacteriocin klebcin, was previously reported to be associated with a phage sequence suggesting its lateral gene transfer and diversification [25].

**Table 2:** Genomic location of PAPE associated with variant BC *lsc* alleles.

PAPE association with	Genomic location	Length (bp)
Prophage PSPPH01, putative cellulase (PSPPH 0655), 1448A chromosome ^#§^	773003–773504	502
putative GH5 Cellulase, (Psyr_4600), B728a chromosome ^#§^	5460116–5459615	502
putative bacteriocin, (PSPTO_0572), DC3000 chromosome ^#§^	629397–629790 *	394
*lsc-1*/*C*, DC3000 chromosome ^#^	1595373–1594873	501
*lsc-3*/*B*, DC3000 plasmid pDC3000A ^#^	34651–34152	500
*lscC* (Psyr_0754), B728a chromosome ^#^	859840–859339	502
*lscB* (PSPPH_A0027), 1448A large plasmid ^#^	22669–22170	500
*lscC* (PSPPH_4994), 1448A chromosome ^#^	5662790–5663289	500
*lscB*, PG4180, plasmid ^#^	-	500
*lscC*, PG4180, chromosome ^#^	-	500
glycosyl hydrolase, PG4180 ^¤^	-	502

PAPE: Phage-associated promoter element; ^*^(57bp upstream to PSPTO_0572). Reference/tool: BLAST-N (#), www.pseudomonas.com (§), This study (¤).

Interestingly, the 48-bp 5’-coding sequences of variant BC *lsc* alleles and the glycosyl hydrolase genes were found to be 80% identical. These 48 nucleotides encode with almost identical N-termini of variant BC *lsc* gene products as well as the putatively pro-phage-borne glycosyl hydrolases (Figure 4). Furthermore, the conserved upstream sequences included the experimentally determined promoter region of variant BC alleles and a phage-associated *com* gene encoding a putative translational regulator [26] (Figure 3). 

The distribution of the ~500-bp conserved upstream and N-terminus-encoding sequences at various genomic positions in diverse *P. syringae* strains suggested their mobile nature (Table 2). Mobile DNA sequences possessing potential promoter regions are generally termed mobile promoter elements [27,28]. These DNA elements, often phage-associated, allow for expression of adjacent genes or re-activation of silent genes such as shown for the IS3-mediated activation of *argE* in *E. coli* [27], and are therefore termed phage-associated promoter elements (PAPE). Interestingly, the herein observed PAPE of *P. syringae* seems to be associated with genes encoding for extra-cellular levansucrases and putative glycosyl hydrolases, both of which might play an important role for nutrient acquisition and *in planta* fitness of the pathogen.

### 2.6. Investigating the Role of *com* Gene and Glycosyl Hydrolase Gene in PG4180

The location of the mapped minimal promoter required for *lsc* expression appeared to be located ≥332-bp upstream from the translational start of the *lscB/C* ORF. This stimulated some interest to scan this inter-genic region for additional genes by comparing this sequence with entries of the GenBank nucleotide sequence database using the method BLAST-N. The search for potential additional coding sequences revealed the presence of a 192-bp ORF starting at position -204 and ending -12 bp upstream of the translational start sites of *lscB* and *lscC*, respectively. This ORF was homologous to the *com* gene [29,30] (Figure 3). Its predicted amino acid sequence exhibited a high degree of similarity to Com translational regulators found in pseudomonad bacteriophages, including Mu-like phage of *P. entomophila* L48 (85.4% similarity), phage B3 of *P. aeruginosa* (78.3% similarity), and phage DVM 2008 of *P. fluorescens Pf-5* (65% similarity). *In-silico* structural prediction at the ‘SUPERFAMILY database of the structural and functional protein website (supfam.mrc-lmb.cam.ac.uk/SUPERFAMILY/index.html) suggested that the predicted *com* gene products possess classic C2-H2 zinc-finger domain as reported for Com earlier [31]. Homologs of the putative *com* gene in PG4180 were also found upstream of the *lsc* and glycosyl hydrolase genes in 1448A, DC3000 and B728a (Figure 2). Moreover, data derived from the www.pseudomonas.com website suggested that the putative phage-borne *cellulase* gene (PSPPH0655) in 1448A and its homolog in B728a (Psyr_4600) are surrounded by phage-associated genes, which with caution suggested potential ancestral pro-phage insertions.

A potential impact of *com* on levan production was investigated by inserting a premature stop codon to *com* using site-directed mutagenesis of plasmid −666-*lscB*. The mutated plasmid was introduced to the levan-deficient mutant PG4180.M6 [3] and compared to a transformant of PG4180.M6 carrying a non-mutated plasmid. Both transformants exhibited similar levels of levan formation and Lsc secretion (Data not shown) demonstrating that the putative translational regulator, Com, was not involved in expression of *lscB*. Its potential role in controlling translation of the glycosyl hydrolase gene needs to be tested in future studies.

**Figure 5:**
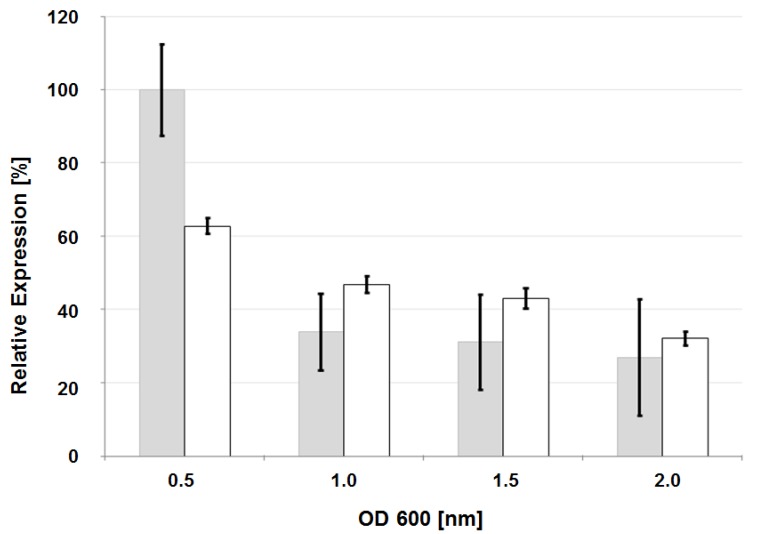
Quantitative Reverse Transcriptase PCR analysis of growth phase-dependent *lsc* and *glycosyl hydrolase* gene expression in *P. syringae* PG4180. Cells were grown at 18 °C in HS + glutamate as sole carbon source. Relative mRNA levels were related to the mean value determined for the signals of *lsc* gene of PG4180 at an OD_600_ of 0.5, which was defined as 100%. Data show the means and standard errors of two experiments with three replicates. Grey bars depicts the expression of *lsc* gene and white bars represent the *glycosyl hydrolase* gene expression in PG4180.

Over-expression of the glycosyl hydrolase gene associated with the herein discovered PAPE in PG4180 and the subsequent protein purification yielded no detectable cellulose-degrading activity [32,33] (Data not shown), suggesting that the encoded enzyme is not a cellulase. A growth-phase dependent transcriptional analysis of the glycosyl hydrolase gene of PG4180 was conducted using qRT-PCR to compare if its expression resembles that of *lsc* as published earlier [4]. Cells of PG4180 wild type were grown in minimal medium containing glutamate at 18 °C. Results indicated that *lsc* and the glycosyl hydrolase gene associated with the PAPE showed maximal expression at the early exponential growth stage (Figure 5). A very similar pattern of expression had previously been observed for the *lscBC* genes [4]. Thus, the result indicated that the PAPE might be the site of transcriptional regulation where common, regulator(s), yet-to-be-identified, might bind and lead to gene expression. 

### 2.7. Putative Scenario for *lsc* Gene Distribution in *P. syringae*

Due to its sequence similarity, its size, and the heterologous expression of the variant A *lsc* gene of PG4180 observed herein, it is tempting to speculate that variant A alleles were initially obtained by horizontal gene transfer from enterobacterial species such as *E. amylovora*. This assumption is fueled by the fact that *P. syringae* is the only known organism, in which multiple copies of *lsc* exist. Consequently, variant A might represent the most ancestral *lsc* variant in *P. syringae*. However, due to its potentially inactive promoter sequence, this variant remained cryptic in *P. syringae*. In an unknown sequence of events initially involving a gene duplication of *lscA*, a pro-phage insertion bearing an active promoter, a potential translational regulator, and the pro-phage-borne N-terminal sequence might have inserted upstream of one of the two variant A gene copies yielding an ancestral variant BC *lsc* copy. Subsequently, transposon-mediated transposition events might have led to a spreading of variant BC copies, now functional, in various *P. syringae* genomes. The latter assumption is indirectly supported by the fact that *P. syringae* pv. tomato PT23 and pv. glycinea race 4 contain multiple copies of IS1240 with the *tnpA* gene coding for a transposase [20,22,34].

Since there are several plasmid-borne variant BC alleles, it is tempting to speculate furthermore that conjugative transfer of *lsc*-bearing plasmids might have led to an accelerated distribution of variant BC alleles among *P. syringae* pathovars. Interestingly, the plasmid-borne *lsc-3* of DC3000 shows an upstream sequence very similar to that of plasmid-borne variant BC alleles of strains PG4180 and 1448A. However, its downstream sequence compares better to the downstream sequences of chromosomal orthologs in strains PG4180 and 1448A, respectively, (Figure 2 and Appendix A2) thus complicating the clarity of further definition of the phylogenetic pathway. However, the presence of two copies of variant BC, of which one is located on a plasmid, might indicate the importance of Lsc for the evolutionary fitness of the leaf pathogen, *P. syringae*.

## 3. Materials and Methods

### 3.1. Bacterial Strains, Plasmids and Growth Conditions

Bacterial strains and plasmids used in this study are listed in Table 3. *Escherichia coli* and *Erwinia amylovora* strains were maintained and grown on Luria-Bertani (LB) medium at 37 °C and 28 °C, respectively [14,35]. *P. syringae* cultures were grown in a modified Hoitink Sinden (HS) medium [36] supplemented with glutamate as sole carbon and nitrogen source at 18 °C. Bacterial growth in liquid LB media was continuously monitored by measuring the optical density at 600 nm (OD_600_) and harvested for protein sampling at an OD_600_ of 1.0. Antibiotics were added to the media at the following concentrations (µg/mL), respectively: ampicillin, 50; kanamycin, 25; tetracycline, 25. Cellulolytic activity was assessed according to Kasana *et al.*, 2008 [32].

### 3.2. Molecular Genetic Techniques

Small scale isolation of plasmid DNA, restriction enzyme digests, agarose gel electrophoresis, purification of DNA fragments from agarose gels, electroporation, ligation of DNA fragments and other routine molecular methods were performed using standard protocols [35]. Nucleotide sequencing was carried out commercially (Eurofins MWG Operon, Ebersberg, Germany). The 3.1-kb *Pst1* fragment containing PG4180 *lscA* was obtained from pSKL3 [3] and re-cloned in pBBR1MCS-3 [37] in the opposite direction to the vector-borne *lac* promoter yielding pBBR3(*lscA*). This construct was subsequently electroporated into competent cells of Ea7/74-LS6 yielding the transformant LS6(*lscA*), which was then grown on LB agar plate containing Km^r^ and Tc^r^. Later, the transconjugant was streaked on 5% sucrose-containing LB agar media. 

**Table 3:** Bacterial strains and plasmids used in this study.

Strain	Relevant characteristics^a^	Reference or source
*Pseudomonas syringae* pv. Glycinea		
PG4180	wild type, levan+	[47]
PG4180.M6	Sp^r^, Gm^r^, *lscB lscC* mutant of PG4180, levan-	[3]
PG4180.M6 (pBBR1MCS-3)	Sp^r^, Gm^r^, Tc^r^, *lscB lscC* mutant of PG4180 bearing pBBR1MCS	This Study
*Erwinia amylovora*		
Ea7/74	Ea7/74	Ea7/74
Ea7/74-LS6	Ea7/74-LS6	Ea7/74-LS6
*Escherichia coli*		
DH5α	*supE*44 Δ*lac*U169 (Φ80 *lacZ*ΔM15) *hsdR*17 *recA*1 *endA*1 *gyrA*96 *thi*-1 *relA*1	[35]
pSKL3	Ap^r^, contains *lscA* on 3.0-kb *Pst*1 insert, (P_lac _> *lscA*)	[3]
pBBR3(*lscA*)	Tc^r^, contains *lscA* on 3.0-kb *Pst*1 insert, (*lscA* > P_lac_)	This study
pBBR1MCS	Cm^r^, broad-host-range cloning vector	[37]
pBBR1MCS-3	Tc^r^, broad-host-range cloning vector	[37]
-666-lscB	Tc^r^, *lscB* gene with -666bp upstream sequence in pBBR1MCS-3	This study
-500-lscB	Tc^r^, *lscB* gene with -500bp upstream sequence in pBBR1MCS-3	This study
-440-lscB	Tc^r^, *lscB* gene with -440bp upstream sequence in pBBR1MCS-3	This study
-332-lscB	Tc^r^, *lscB* gene with -332bp upstream sequence in pBBR1MCS-3	This study
-300-lscB	Tc^r^, *lscB* gene with -300bp upstream sequence in pBBR1MCS-3	This study
-250-lscB	Tc^r^, *lscB* gene with -250bp upstream sequence in pBBR1MCS-3	This study
-200-lscB	Tc^r^, *lscB* gene with -200bp upstream sequence in pBBR1MCS-3	This study
-150-lscB	Tc^r^, *lscB* gene with -150bp upstream sequence in pBBR1MCS-3	This study
-100-lscB	Tc^r^, *lscB* gene with -100bp upstream sequence in pBBR1MCS-3	This study
-50-lscB	Tc^r^, *lscB* gene with -50bp upstream sequence in pBBR1MCS-3	This study
-666-lscC	Tc^r^, *lscC* gene with -327bp upstream sequence in pBBR1MCS-3	This study
-500-lscC	Tc^r^, *lscC* gene with -161bp upstream sequence in pBBR1MCS-3	This study
-440-lscC	Tc^r^, *lscC* gene with -101bp upstream sequence in pBBR1MCS-3	This study
-332-lscC	Tc^r^, *lscC* gene with -332bp upstream sequence in pBBR1MCS-3	This study
-666-lscB.com1	Cm^r^, *lscB* gene with -666 upstream sequence in pBBR1MCS, com gene containing a premature stop codon	This study

^a^ Ap, ampicillin; Km, kanamycin; Tc, tetracycline.

Nested deletion analysis of the upstream region of *lscB* in plasmid pRB7.2 [3] was conducted using the Erase-a-Base^®^ kit (Promega, Madison, USA). For analysis of the *lscC* upstream region, PCR was used to generate products covering the same region as in the deletion constructs of *lscB* (Table 3). PCR products of the *lscC* upstream region were cloned in vector pBBR1MCS-3 (Table 4). All constructs were introduced to *E. coli* DH5α via electroporation and then transferred by tri-parental mating [38] with helper plasmid pRK2013 [39] to the *lscB lscC* mutant PG4180.M6 [3]. Transconjugants were streaked on 5% sucrose-containing MG agar medium for assessment of levan production.

**Table 4:** Oligonucleotide primers used in this study.

Oligonucleotides	Nucleotide sequence (5’-3’) ^a^
lscB_PG-666_fwd	GATGAGCTCCTAAGGCAGTCGCATTAA
lscB_PG-500_fwd	GATGAGCTCAGTCGCAATTAATGCGAG
lscB_PG-440_fwd	GATGAGCTCCCAGGTCAATGGCGCAGC
lscB_PG-332_fwd	GATGAGCTCCACGATATGCGATTTGCG
lscB_PG-300_fwd	GATGAGCTCCCGGATACGGGCTTTTAA
lscB_PG-250_fwd	GATGAGCTCACCCCGCCCAGCCGGGGT
lscB_PG-200_fwd	GATGAGCTCCAAATGTTGAAAGACTAC
lscB_PG-150_fwd	GATGAGCTCCATGGGTGACTACACCGA
lscB_PG-100_fwd	GATGAGCTCTGAATCATGTGAAGGCCG
lscB_PG-50_fwd	GATGAGCTCGGTACACGAGCGTCGCTG
lscB_PG_rev	CGATCTAGATCAGCTTAGCGTCACGTC
lscC_PG-666_fwd	GATGAGCTCAGCTCTGCCAGAAACAGG
lscC_PG-500_fwd	GATGAGCTCTCATAGGAAATTCCTTTT
lscC_PG-440_fwd	GATGAGCTCCCGGGTCAATTGCGCAAC
lscC_PG-332_fwd	GATGAGCTCCACGATATGCGATTTGCG
lscC_PG_rev	CGATCTAGATCAGCTCAGTTGCACGTC
com1	GCAAATGTTGAAAGACTACCGATGCGGGCAGTGC
lscBC_RT_fwd	TCGGTTATCCTGACCCTGAC
lscBC_RT_rev	CCATGACGATCTTCCCAGTC
cel_RT_fwd	ACAAGATGGCCGCTTTATC
cel_RT_rev	TTCGCTTTATCGAGCAGGTT

^a^ Restriction sites incorporated in primers are underlined; GAGCTC—*Sac*I, TCTAGA—*Xba*I.

### 3.3. Extra-Cellular Lsc Detection

Extra-cellular fractions obtained from Ea7/74, Ea7/74-LS6, LS6 (*lscA*), PG4180, and *lsc* deletion constructs-harboring PG4180.M6 transformants and the use of polyclonal antibodies were carried out as described previously [4]. For immunological detection of Lsc enzyme, Western blot experiments were performed with total extra-cellular fractions using polyclonal antibodies raised against purified Lsc of *P. syringae* pv. phaseolicola as described earlier [3]. Water agar plates with 1.5% agar and 5% sucrose were used for the qualitative visualization of extra-cellular Lsc. Lsc activity was quantified by measuring the amount of glucose liberated during incubation with sucrose using the Gluco-quant Glucose/HK assay kit (Roche Diagnostics, Mannheim, Germany) at an absorbance of 340 nm. One unit of Lsc activity corresponded to the amount of enzyme which liberates 1 μmol glucose per minute from sucrose. The experiments were repeated three-fold and mean values were expressed as the quantity of glucose release.

### 3.4. Analysis of *Glycosyl Hydrolase gene* Expression by Quantitative Reverse-Transcriptase Polymerase Chain Reaction (qRT-PCR)

Bacterial cells were grown in HS + glutamate medium at 18 °C. When cultures reached distinct OD_600_ values, total RNA was isolated by acid phenol/chloroform extraction as described previously [40]. The yield and the purity of RNA were determined by measuring absorption at 260 and 280 nm. Total mRNA samples were treated with TURBO DNA-free (Applied Biosystems, Darmstadt, Germany) to remove remaining traces of genomic DNA as described by the manufacturer’s recommendation. 

SYBR green-based qRT-PCR was performed with 1 ng RNA template and 200 nM primers (cel_RT_fwd and cel_RT_rev) (Table 4) using the QuantiTect SYBR Green one-step RT-PCR Kit (Qiagen, Hilden, Germany) according to the manufacturer’s instructions. The thermocycler program comprised an initial step of 95 °C for 15 min followed by 40 cycles of 95 °C for 30 s, 55 °C for 30 s, 72 °C for 30 s. Reactions were performed in technical duplicates and biological triplicates with a Mastercycler^®^ ep realplex2 real-time PCR system (Eppendorf, Hamburg, Germany) as described by the manufacturer using their universal program. Reactions with no addition of reverse transcriptase served as negative controls and proved lack of DNA contamination. Specificity of amplification was assessed by analyzing the melting curve of the amplification product. Due to very high sequence identity between *lscB* and *lscC,* it was not possible to design primers discriminating between these two mRNAs, thereby the expression profile of *lsc* is always referred to as a combination of both genes.

### 3.5. Bioinformatics Analyses

Vector NTI Advance 10.1.1 (Invitrogen Corporation, USA) was used for the nucleotide and amino acid sequence alignments and for dendrogram generation. BLAST-N and BLAST-P programs were used for online sequence analyses and for identifying transposase-like sequences and mobile promoter elements [41]. The website www.pseudomonas.com was consulted for the determination of *P. syringae* gene orthologs and paralogs. 

## 4. Conclusions and Future Scope

Due to the high degree of conservation we can hypothesize that the conserved PAPE, identified herein, appears to be involved in the expression of variant BC *lsc* alleles and the glycosyl hydrolase gene in a coordinated manner. The sequence of the PAPE might harbor binding sites for regulatory proteins possibly controlling sugar utilization in *P. syringae*. This exciting hypothesis is fueled by the idea that *P. syringae* might need to produce both sucrose-utilizing Lsc and plant cell wall-degrading glycosyl hydrolase, in order to obtain glucose for central metabolism. Phage-mediated genetic rearrangements might have made possible such a coordinated control of the gene products of sugar metabolism. It is tempting to speculate that this type of regulation might be linked to that of central cellular sugar utilization. Thus, future experiments will focus on analyzing the potentially coordinated expression of variant BC *lsc* genes, the glycosyl hydrolase gene, and other genes required for central glucose metabolism. Likewise, identification of the enzymatic function of the gene product encoded by the glycosyl hydrolase gene, discovered herein, will be part of our future research focus. Occurrence of three isoforms of Lsc in *P. syringae* indicates their importance in this plant pathogenic bacterium. The precise additional roles of levan formation aside from nutrient acquisition, adherence to plant surfaces, or as protective functions remain to be determined.
